# Supplementary material for: Identification of photoperiod-induced specific miRNAs in the adrenal glands of Sunite sheep (Ovis aries)
Source: Front Vet Sci. 2022 Jul 22;9:888207. doi: 10.3389/fvets.2022.888207 (PMC9354845; doi:10.3389/fvets.2022.888207)
Supplement: Supplementary file 1 [file Data_Sheet_1.ZIP › Supplementary file/miRDeep2 file.zip!/miRDeep2 file/Novel_Structure/LP42A1/Novel_475.pdf]

Provisional ID	:	NC_019471.2_23356
Score total	:	16.7
Score for star read(s)	:	3.9
Score for read counts	:	9.8
Score for mfe	:	1.4
Score for randfold	:	1.6
Score for cons. seed	:	
Total read count	:	31
Mature read count	:	30
Loop read count	:	0
Star read count	:	1

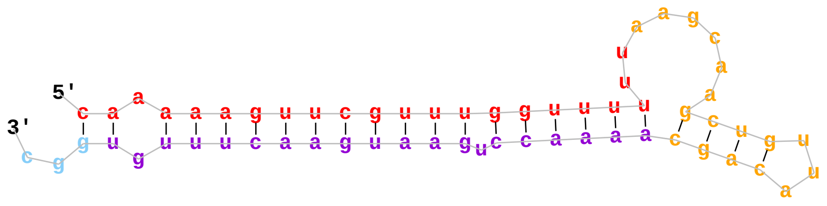

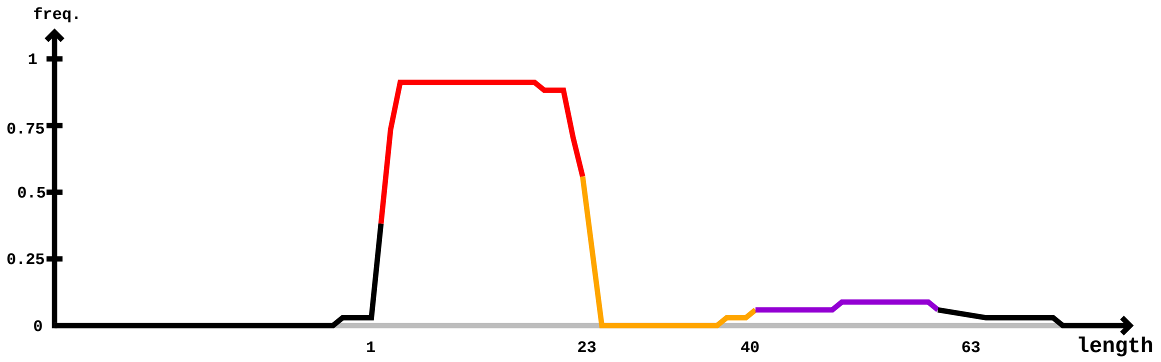

Mature Star

```
5'- acgcuuccccucugauacacuacugggcuggccaaaaaguucguuugguuuuuuaagcaagcuguuacagcaaaaccugaaugaacuuuguggccaacccaagacucugu  -3'  obs
    acgcuuccccucugauacacuacugggcuggccaaaaaguucguuugguuuuuuaagcaagcuguuacagcaaaaccugaaugaacuuuguggccaacccaagacucugu       exp
    .......................((((.((((((.(((((((((((((((((........((((...)))))))))).))))))))))).)))))).)))).........  reads  mm  sample
    ............................uggccaaaaaguucguuuggA.............................................................  1      1   seq
    ................................caaaaaguucguuuggGuuuu.........................................................  5      1   seq
    ................................caaaaaguucguuuggGuuuuu........................................................  7      1   seq
    .................................aaaaaguucguuuggGuuu..........................................................  6      1   seq
    .................................aaaaaguucguuugguuuuuu........................................................  1      0   seq
    .................................aaaaaguucguuuggGuuuuua.......................................................  5      1   seq
    ..................................aaaaguucguuuggGuuuuua.......................................................  6      1   seq
    ....................................................................agAaaaaccugaaugaacuuug....................  1      1   seq
    .......................................................................aaaaccugaaugaacuuugu...................  1      0   seq
    ................................................................................augaacuuuUuggccaacccaag.......  1      1   seq
```
